# Neck Muscle Changes Following Long-Duration Spaceflight

**DOI:** 10.3389/fphys.2019.01115

**Published:** 2019-09-13

**Authors:** Kyle P. McNamara, Katelyn A. Greene, Janet A. Tooze, Jade Dang, Karim Khattab, Leon Lenchik, Ashley A. Weaver

**Affiliations:** ^1^Department of Biomedical Engineering, Center of Injury Biomechanics, Wake Forest School of Medicine, Winston-Salem, NC, United States; ^2^School of Biomedical Engineering and Sciences (SBES), Virginia Tech – Wake Forest University, Winston-Salem, NC, United States; ^3^Department of Biostatistical Sciences, Wake Forest School of Medicine, Winston-Salem, NC, United States; ^4^Department of Radiology, Wake Forest School of Medicine, Winston-Salem, NC, United States

**Keywords:** advanced resistive exercise device, astronaut, cosmonaut, cross-sectional area, cervical muscle, microgravity, muscle hypertrophy, magnetic resonance imaging

## Abstract

The effects of long-duration spaceflight on crewmember neck musculature have not been adequately studied. The purpose of this study was to evaluate the changes in the neck musculature on pre-flight and post-flight magnetic resonance imaging (MRI) examinations of six crewmembers on 4- to 6-month missions equipped with the advanced resistive exercise device (aRED). The MRI images were resliced to remove variations in spinal curvature, the cross-sectional area (CSA), and muscle fat infiltration (MFI) of neck musculature at the C1-C2, C4-C5, C7-T1, and T1-T2 intervertebral disc levels were measured bilaterally. Percent changes in the neck muscle CSA and fatty infiltration following spaceflight were calculated, and mixed models were used to assess significance of these changes. Crewmembers on missions equipped with the aRED experienced an average 25.1% increase in CSA for the trapezius muscle at C6-C7, an average 11.5% increase in CSA for the semispinalis capitis muscle at C4-C5, an average 9.0% increase in CSA for the sternocleidomastoid muscle at C4-C5, and an average 23.1% increase in CSA for the rhomboid minor at T1-T2. There were no significant changes in the CSA of the levator scapulae, splenius capitis, rectus capitis posterior major, scalenus anterior, scalenus posterior, scalenus medius, longissimus capitis, or obliquus capitis inferior muscles at the locations measured. None of the muscles analyzed experienced statistically significant changes in fatty infiltration with spaceflight. Our study indicates that long-duration spaceflight conditions are associated with preservation of CSA in most neck muscles and significant increases in the CSAs of the trapezius, semispinalis capitis, sternocleidomastoid, and rhomboid minor muscles. This may indicate that cervical muscles are not subjected to the same degradative effects microgravity imparts on the majority of muscles.

## Introduction

Lower extremity skeletal muscle atrophy has been established for crewmembers on long-duration spaceflights (LeBlanc et al., 2000a; Ploutz-Snyder et al., 2015). The risk of injury to astronauts while on mission, upon landing, and after returning to gravity can be increased as a result of microgravity induced muscle degradation (LeBlanc et al., 1998, 2000a,b; Adams et al., 2003; Lang et al., 2004; Ploutz-Snyder et al., 2015). One common spinal injury in astronauts is a herniated nucleus pulposus (i.e., herniated disc). Disc herniations occur 4.3 times more often in astronauts’ lifetimes, with the incidence of herniation in the cervical spine being 21.4 times higher than that of the civilian population (Johnston et al., 2010). The immediate risk of disc herniation upon return from mission is even higher; astronauts are 35.9 times more likely to experience a herniation during the first year post-flight (Johnston et al., 2010). Recent studies have found an association between spinal muscle atrophy and decreased lumbar lordosis that can lead to disc herniation (Bailey et al., 2018; Green and Scott, 2018).

Prior studies have not reported any significant changes in overall neck musculature following long-duration spaceflight (LeBlanc et al., 2000a). However, there is limited information on the changes of individual neck muscles and the effects of current in-flight exercise interventions on the neck musculature. Bed rest studies have been used to investigate physiological effects of microgravity, given the ease of conducting tests, and the ability to recruit larger sample sizes. One such study focusing on neck muscle changes after 60 days of bed rest found significant increases in muscle volume for the splenius capitis, spinalis cervicis, longus capitis, longus colli, levator scapulae, sternocleidomastoid, and all three scalenes (Belavý et al., 2013). However, it has been postulated that differences in cervical movements during spaceflight compared to bed rest may lead to differences in spinal muscle changes between the two groups (LeBlanc et al., 1992). Therefore, studies of spaceflight spinal musculature are needed to supplement and validate the results of bed rest studies.

It is important to note that muscle atrophy/hypertrophy is only one component affecting crewmember injury risk following long-duration spaceflight. The combination of spaceflight-induced loss of muscle strength, sensorimotor impairment, reduced postural stability, and bone loss contributes to injury during the dynamic loading encountered in spacecraft landings, post-flight falls, and in daily living (LeBlanc et al., 2000a; Lang et al., 2004; Muir et al., 2011; Wood et al., 2011). To address these concerns, in 2008 the International Space Station (ISS) mission 18 introduced an advanced resistive exercise device (aRED) to simulate earth-based gravitational pull and mimic weight-bearing exercise. Previous studies have investigated the effects of long-duration spaceflight on paraspinal muscles, reporting decreased cross-sectional area (CSA) measurements in the erector spinae, multifidus, and quadratus lumborum muscles (Burkhart et al., 2018; McNamara et al., 2019). However, bed rest studies suggest that these beneficial effects may not translate to the cervical region (Belavý et al., 2013).

Magnetic resonance imaging (MRI) is a commonly used imaging modality for measuring the size and composition of the neck muscles (Elliott et al., 2005; Belavý et al., 2013). Performing these measurements both before and after long-duration spaceflight allows for the investigation of the effects of microgravity on overall muscle health. The primary objective of this study was to assess pre- to post-flight changes in the neck muscle CSA on cervical MRI images of crewmembers (*n* = 6) on 4- to 6-month aRED-equipped missions. The initial hypothesis was that neck muscles would undergo declines in CSA as a result of prolonged microgravity. In addition, measures of muscle composition were explored as a secondary analysis. It was hypothesized that neck muscles would experience greater levels of fatty infiltration with long-duration spaceflight.

## Materials and Methods

The mission durations, pre- and post-flight MRI scans and isokinetic data, and in-flight exercise training logs were obtained from the National Aeronautics and Space Administration (NASA) Life Sciences Data Archive and Lifetime Surveillance of Astronaut Health project. Written informed consent was obtained from each crewmember and the study protocols were approved by the institutional review boards at the Wake Forest School of Medicine and at NASA. Pre- and post-flight T1-weighted MRI scans of the cervical regions were acquired using a Siemens Magnetom Verio 3T scanner for six crewmembers (average age, 48 ± 4.8 years) on expeditions lasting 166 ± 14.8 days in duration (NASA, 2017).

**FIGURE 1:**
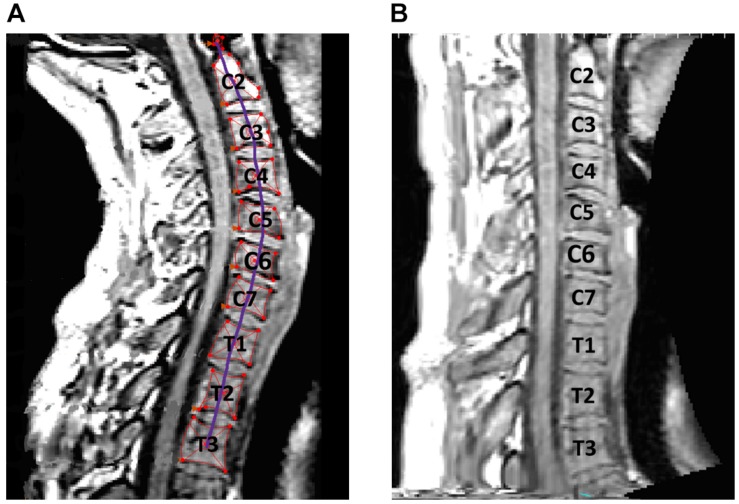
**(A)** Centroids for each cervical vertebra were determined. **(B)** Spline curve through centroids was used to produce a re-sliced scan that standardized neck curvature across subjects.

To ensure accurate comparison of pre- and post- flight neck muscle CSA, consistent measurement location was used. Our image analysis protocol accounted for inconsistencies in lordotic curvature during patient positioning. Moreover, a decrease in lordotic curvature has been associated with spaceflight, and our measurement techniques were adjusted to correct for these changes (Stemper et al., 2010). Prior studies have made these corrections by aligning the axial view parallel to each intervertebral disc during scan acquisition (Stemper et al., 2010). Since the MRI scans were collected retrospectively in our study, we employed a method for reslicing the MRI scans, using Mimics software (v20, Materialise, Leuven, Belgium), to allow for consistent measurements. The Mimics reslicing tool axially resliced the MRI images in the direction of a user defined curve. To orient the axial view parallel to the intervertebral discs, a curve was created in the sagittal view which passed through the centroids of each vertebra and traveled perpendicularly through the intervertebral discs (Figure 1A). When resliced along this curve, the newly created axial view aligned parallel to the intervertebral discs at every vertebral level (Figure 1B). To ensure a consistent reslicing method between scans, the resliced curve was generated using the sagittal slice cutting through the center of the spinal cord at the C2-C3 intervertebral disc level. This vertebral level was chosen since the retrospective MRI scans were originally aligned parallel to the C2-C3 intervertebral disc per the scanning protocol.

After reslicing the scans, the neck muscle CSA measurements were collected using the Mimics measurement tool. This tool uses an algorithm based on Hounsfield Unit values to interpolate muscle boundaries between manually selected points. Several points were chosen along the perimeter of each neck muscle to create a smooth, accurate muscle boundary and determine muscle CSA.

**FIGURE 2:**
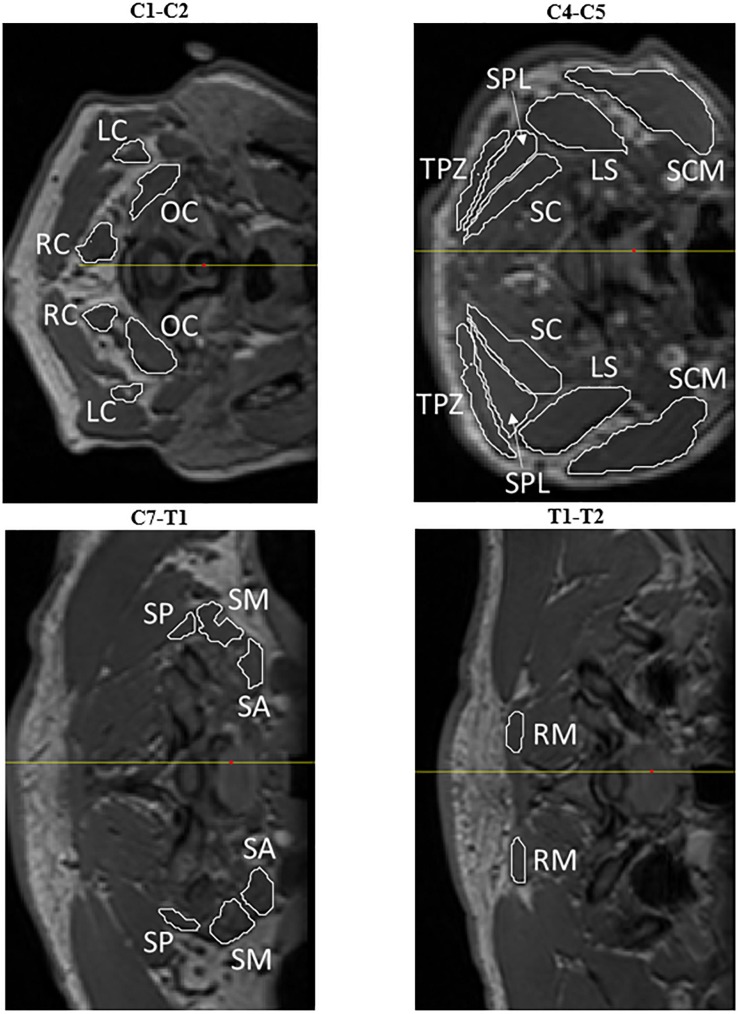
Cross-sectional measurements were acquired at specific intervertebral levels for the longissimus capitis (LC), obliquus capitis inferior (OC), rectus capitis posterior major (RC), sternocleidomastoid (SCM), levator scapulae (LS), trapezius (TPZ), semispinalis capitis (SC), splenius (SPL), scalenus posterior (SP), scalenus medius (SM), scalenus anterior (SA), and rhomboid minor (RM).

To ensure accurate comparison of muscle CSA from pre- to post-flight scans, the vertebral level at which the measurements were made was based on previous literature (Elliott et al., 2007; Fernandez-de-Las-Penas et al., 2007; Oksanen et al., 2008; Stemper et al., 2010). Muscles not measured in previous studies were measured at the same vertebral level as the neighboring muscles or at the most inferior intervertebral disc present on all scans (T1-T2). Twelve cervical muscles were measured at one of four measurement locations shown in Figure 2 and Table 1. Measurements were made on both the right and left sides of each muscle, providing a total of 12 pre-flight and 12 post-flight samples for each muscle across the six crewmembers. The mean pre- to post-flight percent change for each muscle was calculated by summing the individual changes and dividing by the sample size (*n* = 12).

**TABLE 1:** Vertebral level of muscle cross-sectional area (CSA) measurements.

**Measurement location**	**Muscles measures**
C1-C2 intervertebral disc	Longissimus capitis, obliquus capitis inferior, rectus capitis posterior major (Elliott et al., 2007)
C4-C5 intervertebral disc	Levator scapulae (Oksanen et al., 2008; Stemper et al., 2010), semispinalis capitis (Elliott et al., 2007; Oksanen et al., 2008), splenius (combined capitis and cervicis) (Elliott et al., 2007; Oksanen et al., 2008; De Loose et al., 2009), sternocleidomastoid (Oksanen et al., 2008; Stemper et al., 2010), and trapezius (Elliott et al., 2007; Oksanen et al., 2008; Stemper et al., 2010)
C7-T1 intervertebral disc	Scalenus anterior (De Loose et al., 2009), scalenus medius (De Loose et al., 2009), and scalenus posterior (De Loose et al., 2009)
T1-T2 intervertebral disc	Rhomboid minor

**FIGURE 3:**
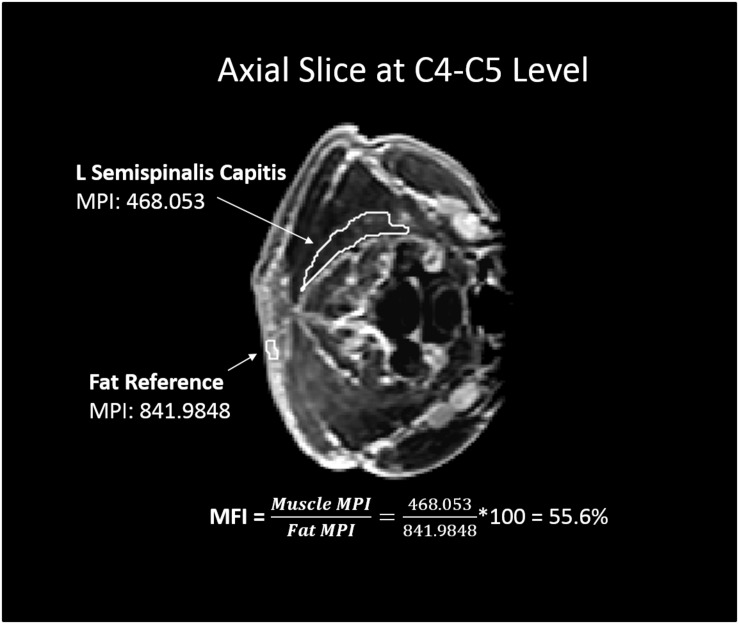
Example of muscle fat infiltration (MFI) calculation. White outlined regions of interest represent a muscle (left semispinalis capitis) and subcutaneous fat reference used for the MFI measurement at the C4-C5 vertebral level. The resulting MFI calculation is shown, in which the mean pixel intensity (MPI) of muscle was reported as a percentage of the reference fat MPI.

Muscle fat infiltration (MFI) was also measured in Mimics (Figure 3). Muscles were segmented in a single slice, creating regions of interests for each muscle at the same vertebral level as the aforementioned CSA measurement. The segmented pixels across the CSA of each muscle were then automatically aggregated and the mean pixel intensity (MPI) extracted. At each examined vertebral level, a selection of subcutaneous fat was also analyzed and the MPI extracted. The MFI was then reported as a ratio of muscle MPI to fat MPI multiplied by 100 to yield an estimate of percent fat content in the muscle, as shown in Equation 1 (Elliott et al., 2005; Valentin et al., 2015).

(1) $$M\,F\,I=\frac{M\,P\,I_{m\,u\,s\,c\,l\,e}}{M\,P\,I_{f\,a\,t\,r\,e\,f\,e\,r\,e\,n\,c\,e}}*100$$

Statistical analysis was performed using SAS (Version 9.4. SAS Institute Inc., Cary, NC, United States) using a 0.05 level of significance. Shapiro-Wilk w tests were performed to assess the distribution of data. To account for the repeated measures by subject and side, for each of the outcomes of interest (CSA and MFI), mixed models with a random side effect with an unstructured covariance matrix were used to model changes from pre- to post-spaceflight, and allowing for separate residual variance for each muscle type. This model allows for correlation between measures on the same side and between the left and right sides for each subject. Comparisons between the neck muscle CSA changes in this study and historical volumetric bed rest studies with resistive exercise were performed according to the calculations outlined by Whitley and Ball (2002) to compare two means arising from unpaired data.

A subset of four crewmembers had in-flight exercise logs that documented their time using the cycle ergometer with vibration isolation and stabilization system (CEVIS), time using a treadmill, as well as frequency of aRED usage. These crewmembers also had pre- and post-functional fitness assessments consisting of peak trunk flexion torque. Crewmembers also had pre- and post-fitness assessments for maximum pushups, situps, and pullups performed in two minutes, maximum fingertip distance during a sit and reach exercise, and maximum weight lifted while performing smith bench presses and leg presses. No logs of in-flight nutrition were available in this subset.

Shapiro-wilk tests were performed on all exercise frequencies and trunk flexion torque changes to evaluate for normally distributed results. Trunk flexion torque was individually linearly regressed against lumbopelvic muscle changes to assess for any trunk flexion torque changes as a result of radiologically observed findings. Lumbopelvic muscle changes with normal distributions were individually linearly regressed against CEVIS (min/day), treadmill (min/day), and aRED (uses/day) to evaluate for significant changes resulting from crewmembers’ choice of in-flight fitness routine. These values were determined by taking the total minutes spent using CEVIS or treadmill and total flight use of aRED and normalizing by the mission duration in days.

## Results

**FIGURE 4:**
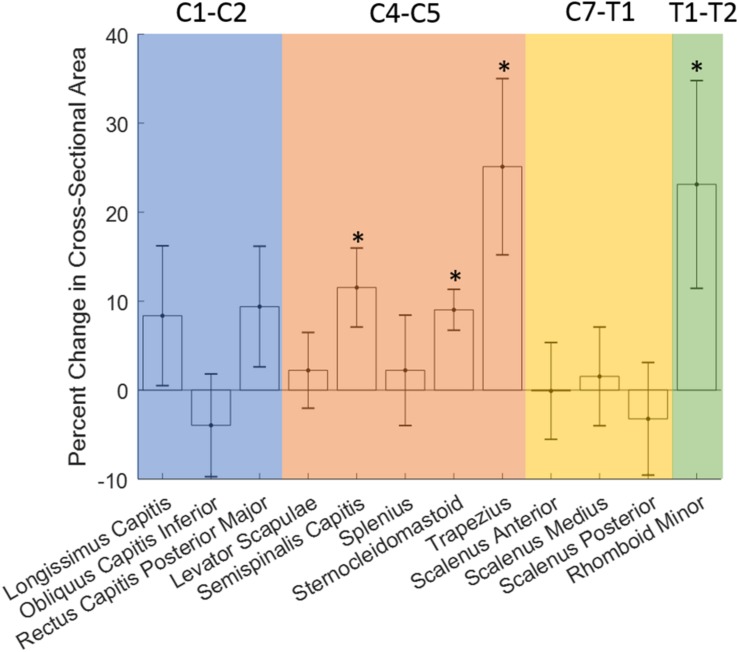
Changes in neck muscle cross-sectional area (CSA) with long-duration spaceflight. Error bar denotes standard error. ^∗^Denotes significance with *p* < 0.05.

Pre- to post-flight changes in the muscle CSA are shown in Figure 4 and Table 2. The Shapiro-Wilk w test confirmed a normal distribution of all neck muscle changes (*p* > 0.05). Post-flight increases in muscle CSA were observed in the semispinalis capitis (mean: 11.5%, SE: 4.4%, *p* = 0.0106), sternocleidomastoid (mean: 9.0%, SE: 2.3%, *p* = 0.0001), trapezius (mean: 25.1%, SE: 9.9%, *p* = 0.0125), and the rhomboid minor (mean: 23.1%, SE: 11.7%, *p* = 0.0500).

**TABLE 2:** Absolute (mm^2^) and percent changes in muscle cross-sectional area (CSA) for the six crewmembers (Subject 1–Subject 6).

**Disc location**	**Muscle**	**Subject 1**						**Subject 2**						**Subject 3**					
		**Left**			**Right**			**Left**			**Right**			**Left**			**Right**		
		**Pre**	**Post**	**%**	**Pre**	**Post**	**%**	**Pre**	**Post**	**%**	**Pre**	**Post**	**%**	**Pre**	**Post**	**%**	**Pre**	**Post**	**%**
C1-C2	Longissimus capitis	73.6	90.4	22.9	85.6	98.0	14.4	88.8	89.2	0.5	65.3	82.7	26.7	82.9	69.3	–16.4	53.1	69.0	30.0
	Obliquus capitis inferior	99.8	93.5	–6.4	91.0	95.0	4.3	177.2	203.1	14.6	227.9	174.0	–23.7	416.2	390.8	–6.1	275.9	274.9	–0.4
	Rectus capitis posterior major	148.0	145.8	–1.5	127.7	128.0	0.2	145.0	126.7	–12.6	83.2	107.3	29.0	97.6	152.7	56.5	103.3	131.6	27.4
C4-C5	Levator scapulae	258.1	283.8	9.9	240.2	328.2	36.6	334.5	314.2	–6.1	366.7	392.3	7.0	625.3	599.6	–4.1	607.9	626.8	3.1
	Semispinalis capitis	333.7	389.2	16.6	396.0	350.1	–11.6	202.1	232.6	15.1	254.3	305.0	19.9	235.7	275.9	17.1	296.9	335.8	13.1
	Splenius capitis	244.1	252.8	3.6	191.1	222.7	16.5	168.3	214.1	27.2	221.0	275.2	24.5	274.5	213.8	–22.1	325.6	294.6	–9.5
	Sternocleido-mastoid	364.3	419.5	15.2	408.2	448.7	9.9	519.7	545.2	4.9	463.5	589.7	27.2	634.1	678.8	7.0	555.3	557.3	0.4
	Trapezius	260.1	297.0	14.2	225.7	318.7	41.2	135.1	151.2	11.9	200.6	195.1	–2.7	777.8	1149.0	47.7	1090.0	1377.6	26.4
C7-T1	Scalenus anterior	202.9	184.9	–8.9	152.2	191.3	25.6	116.4	119.1	2.3	153.4	150.3	–2.0	231.6	161.5	–30.3	128.9	171.5	33.0
	Scalenus medius	173.6	163.5	–5.8	169.0	135.6	–19.8	169.8	168.6	–0.7	169.8	184.0	8.4	109.8	151.1	37.6	104.8	125.9	20.2
	Scalenus posterior	296.1	126.1	–57.4	127.5	145.8	14.4	57.6	60.1	4.4	69.7	76.5	9.8	110.0	86.3	–21.5	66.4	62.4	–6.0
T1-T2	Rhomboid minor	42.4	71.1	35.0	72.1	133.9	85.8	101.7	185.2	82.1	110.6	185.1	67.4	245.2	181.9	–25.8	223.7	178.8	–20.1

**Disc location**	**Muscle**	**Subject 4**						**Subject 5**						**Subject 6**					
		**Left**			**Right**			**Left**			**Right**			**Left**			**Right**		
		**Pre**	**Post**	**%**	**Pre**	**Post**	**%**	**Pre**	**Post**	**%**	**Pre**	**Post**	**%**	**Pre**	**Post**	**%**	**Pre**	**Post**	**%**

C1-C2	Longissimus capitis	42.8	40.3	–5.9	58.9	32.0	–45.7	83.7	80.8	–3.5	71.2	75.7	6.3	25.9	44.5	71.6	56.6	55.7	–1.6
	Obliquus capitis inferior	126.8	160.0	26.2	205.1	232.6	13.4	348.9	314.5	–9.8	352.9	260.4	–26.2	404.8	301.3	–25.6	427.0	389.2	–8.9
	Rectus capitis posterior major	82.1	97.1	18.3	109.2	72.2	–33.9	88.9	99.7	12.1	106.4	141.6	33.1	147.0	132.6	–9.8	139.5	129.4	–7.2
C4-C5	Levator scapulae	171.8	162.7	–5.3	149.8	141.8	–5.3	205.0	248.9	21.4	245.8	233.5	–5.0	476.7	404.8	–15.1	449.4	397.3	–11.6
	Semispinalis capitis	170.2	178.7	5.0	180.1	195.0	8.3	388.4	374.3	–3.6	401.8	450.4	12.1	222.2	325.6	46.5	479.9	474.4	–1.1
	Splenius capitis	174.8	170.3	–2.5	344.2	263.6	–23.4	289.4	408.8	41.3	320.5	336.8	5.1	398.6	252.5	–36.7	366.5	373.3	1.9
	Sternocleido-mastoid	321.4	343.7	6.9	379.3	338.3	–10.8	539.4	612.7	13.6	623.4	706.9	13.4	517.0	580.4	12.3	592.9	636.6	7.4
	Trapezius	107.9	117.3	8.7	87.9	128.4	46.1	251.4	402.3	60.0	242.4	463.5	91.2	320.7	227.9	–28.9	263.8	222.7	–15.6
C7-T1	Scalenus anterior	98.0	99.3	1.3	110.9	101.0	–8.9	156.4	175.8	12.4	143.3	137.4	–4.1	144.1	109.9	–23.8	110.7	111.9	1.0
	Scalenus medius	151.2	155.2	2.6	113.8	94.1	–17.4	99.3	118.6	19.4	129.4	145.4	12.4	146.1	104.2	–28.7	105.2	94.0	–10.7
	Scalenus posterior	86.0	76.2	–11.4	74.7	80.6	7.9	173.0	200.4	15.8	189.7	226.4	19.3	98.0	85.1	–13.1	213.9	210.1	–1.8
T1-T2	Rhomboid minor	60.0	60.3	0.4	98.4	91.0	–7.6	243.4	185.3	–23.9	200.5	195.2	–2.6	133.3	172.2	29.2	128.9	159.6	23.8

*Absolute and percent changes in Muscle CSA pre- to post-flight.*

Three of six crewmembers had increases in CSA for both semispinalis capitis muscles following spaceflight ranging from 5.0 to 19.9%. The remaining three crewmembers had one semispinalis capitis muscle with an increase and one with a decrease in CSA following spaceflight of −1.1 to −11.6% on one side, and increases from 12.1 to 46.5% on the other side.

Five of the six crewmembers had increases in CSA for both sternocleidomastoid muscles following spaceflight ranging from 0.4% to 15.2%. The remaining crewmember had one sternocleidomastoid muscle with an increase (6.9%) and one with a decrease (−10.8%) in CSA following spaceflight.

Four of the six crewmembers (67%) had increases in CSA for both trapezius muscles following spaceflight ranging from 8.7 to 91.2%. One crewmember had one trapezius muscle with an increase (11.9%) and one with a decrease (−2.7%) in CSA following spaceflight. One crewmember had decreases in CSA for both trapezius muscles following spaceflight ranging from −15.6 to −28.9%.

Three of the six crewmembers had increases in CSA for both rhomboid minor muscles following spaceflight ranging from 23.8 to 85.8%. Two of the six crewmembers had decreases in CSA for both rhomboid minor muscles following spaceflight ranging from −2.6 to −25.8%. One crewmember had one trapezius muscle with an increase (0.4%) and one with a decrease (−7.6%) in CSA following spaceflight.

Neck muscles with non-significant CSA changes included the longissimus capitis (mean: 8.4%, 7.9% SE), obliquus capitis inferior (mean: −4.0%, 5.8% SE), rectus capitis posterior major (mean: 9.4%, 6.8% SE), levator scapulae (mean: 2.2%, 4.3% SE), splenius capitis (mean: 2.2%, 6.2% SE), scalenus anterior (mean: −0.1%, 5.4% SE), scalenus medius (mean: 1.5%, 5.5% SE), and scalenus posterior (mean: −3.2%, 6.3% SE).

Linear fits of the CSA changes in sternocleidomastoid vs. trapezius, sternocleidomastoid vs. semispinalis capitis, sternocleidomastoid vs. rhomboid minor, trapezius vs. semispinalis capitis, trapezius vs. rhomboid minor, and semispinalis capitis vs. rhomboid minor all resulted in statistically non-significant trends (α = 0.05).

**FIGURE 5:**
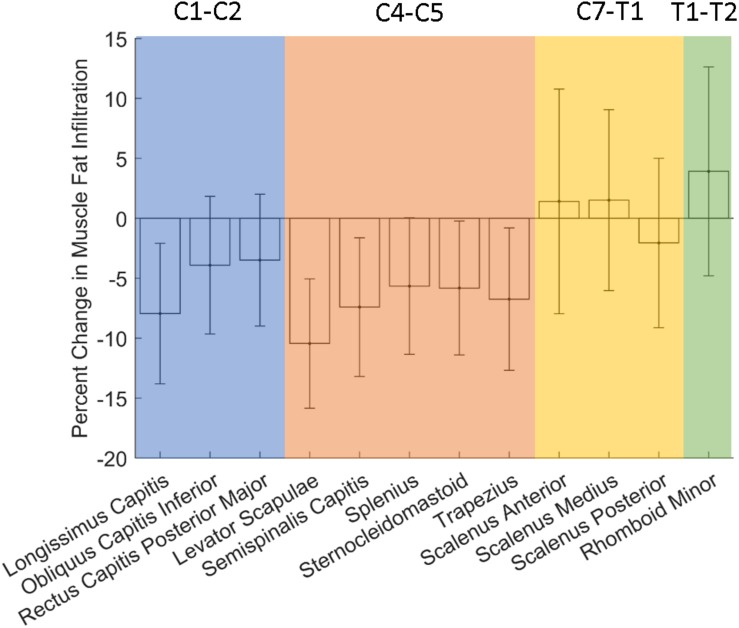
Changes in neck MFI with long-duration spaceflight. Error bar denotes standard error.

None of the muscles analyzed showed stastistically significant changes in fatty infiltration with spaceflight (Figure 5 and Table 3).

**TABLE 3:** Absolute and percent changes in muscle fat infiltration (MFI) for the six crewmembers (Subject 1–Subject 6).

**Disc location**	**Muscle**	**Subject 1**						**Subject 2**						**Subject 3**					
		**Left**			**Right**			**Left**			**Right**			**Left**			**Right**		
		**Pre**	**Post**	**%**	**Pre**	**Post**	**%**	**Pre**	**Post**	**%**	**Pre**	**Post**	**%**	**Pre**	**Post**	**%**	**Pre**	**Post**	**%**
C1-C2	Longissimus Capitis	49.5	37.9	–23.3	59.2	42.9	–27.5	39.6	42.2	6.5	45.2	44.3	–2.0	46.1	44.3	–4.0	43.8	45.0	2.7
	Obliquus Capitis inferior	45.6	33.8	–26.0	50.4	37.9	–24.8	36.5	40.3	10.3	38.8	40.0	3.1	46.2	43.0	–6.9	43.5	43.6	0.2
	Rectus capitis posterior major	50.9	37.2	–26.8	58.6	39.3	–33.0	34.0	37.3	9.7	37.2	40.5	8.8	44.5	44.8	0.6	42.5	46.7	10.0
C4-C5	Levator scapulae	50.5	33.3	–34.0	51.0	30.5	–40.2	41.6	41.6	–0.2	38.7	35.7	–7.6	49.6	47.4	–4.4	40.1	40.9	2.0
	Semispinalis capitis	55.6	36.0	–35.2	62.2	40.7	–34.6	43.3	40.6	–6.2	48.1	42.8	–11.0	45.9	48.9	6.7	46.6	50.8	9.1
	Splenius capitis	49.3	35.1	–28.8	66.8	44.5	–33.4	41.8	40.8	–2.5	51.1	47.0	–8.1	46.4	49.7	7.3	49.9	58.0	16.1
	Sternocleido-mastoid	57.9	38.3	–33.9	48.4	29.0	–40.1	47.0	45.9	–2.5	34.4	31.6	–7.9	54.5	56.2	3.3	37.1	38.8	4.6
	Trapezius	48.0	32.8	–31.5	70.0	40.9	–41.6	43.5	41.4	–4.9	53.2	44.7	–16.0	50.3	51.2	1.7	49.4	59.1	19.7
C7-T1	Scalenus anterior	52.9	32.4	–38.6	48.1	26.8	–44.3	44.6	41.6	–6.7	36.1	32.5	–10.2	43.4	33.1	–23.8	39.0	29.7	–23.8
	Scalenus medius	64.5	38.5	–40.3	54.3	32.1	–40.8	43.8	44.6	1.9	35.1	31.9	–9.2	47.6	40.3	–15.2	34.6	31.5	–9.2
	Scalenus posterior	53.1	37.0	–30.2	53.8	32.8	–38.9	43.1	42.5	–1.6	36.3	31.8	–12.3	46.0	35.7	–22.4	30.2	27.0	–10.6
T1-T2	Rhomboid minor	48.2	42.9	–11.1	54.6	49.9	–8.7	43.7	40.6	–7.0	48.1	47.0	–2.2	50.3	50.0	–0.5	44.3	53.3	20.3

**Disc location**	**Muscle**	**Subject 4**						**Subject 5**						**Subject 6**					
		**Left**			**Right**			**Left**			**Right**			**Left**			**Right**		
		**Pre**	**Post**	**%**	**Pre**	**Post**	**%**	**Pre**	**Post**	**%**	**Pre**	**Post**	**%**	**Pre**	**Post**	**%**	**Pre**	**Post**	**%**

C1-C2	Longissimus capitis	38.7	37.7	–2.6	43.6	42.2	–3.3	41.1	36.9	–10.1	40.7	38.9	–4.2	38.9	31.8	–18.4	45.6	43.4	–4.8
	Obliquus capitis inferior	41.5	39.1	–5.8	38.8	40.1	3.2	39.7	37.3	–6.0	40.8	35.6	–12.6	32.4	38.4	18.6	39.2	40.7	4.1
	Rectus capitis posterior major	40.4	39.8	–1.6	41.3	43.3	4.8	39.8	37.0	–7.0	40.8	38.5	–5.7	36.9	37.2	0.7	41.4	42.3	2.1
C4-C5	Levator scapulae	41.0	37.6	–8.3	39.7	39.3	–1.1	46.2	38.8	–16.0	35.9	34.5	–3.6	43.6	44.2	1.5	46.7	42.5	–9.0
	Semispinalis capitis	39.2	38.4	–2.1	44.2	42.5	–3.8	45.8	36.2	–20.9	44.9	39.9	–11.3	38.1	42.3	11.0	43.1	49.1	13.9
	Splenius capitis	42.9	39.4	–8.1	49.9	48.2	–3.6	43.4	37.6	–13.5	43.6	38.9	–10.9	39.2	43.3	10.6	52.4	58.3	11.4
	Sternocleido-mastoid	45.6	45.7	0.2	34.9	35.0	0.3	46.6	44.0	–5.6	34.5	37.4	8.3	45.0	50.3	11.7	40.3	38.8	–3.7
	Trapezius	42.6	39.1	–8.3	42.8	51.8	20.9	46.9	40.6	–13.6	48.3	45.4	–6.0	41.8	42.0	0.5	59.0	60.5	2.6
C7-T1	Scalenus anterior	38.6	43.9	13.9	29.5	37.8	28.0	37.9	40.0	5.5	37.8	42.7	13.0	25.6	40.4	57.6	24.2	36.5	50.8
	Scalenus medius	41.1	49.8	21.1	29.1	38.3	31.7	41.9	43.0	2.5	30.2	35.8	18.7	32.7	46.4	41.8	31.8	38.0	19.6
	Scalenus posterior	39.3	47.7	21.6	33.9	41.2	21.6	40.6	39.9	–1.9	30.4	35.7	17.6	36.5	46.9	28.2	34.3	37.2	8.5
T1-T2	Rhomboid minor	41.8	44.4	6.2	47.7	51.0	6.9	51.1	35.8	–29.9	52.1	39.4	–24.4	33.8	46.0	36.2	32.0	52.9	65.3

**FIGURE 6:**
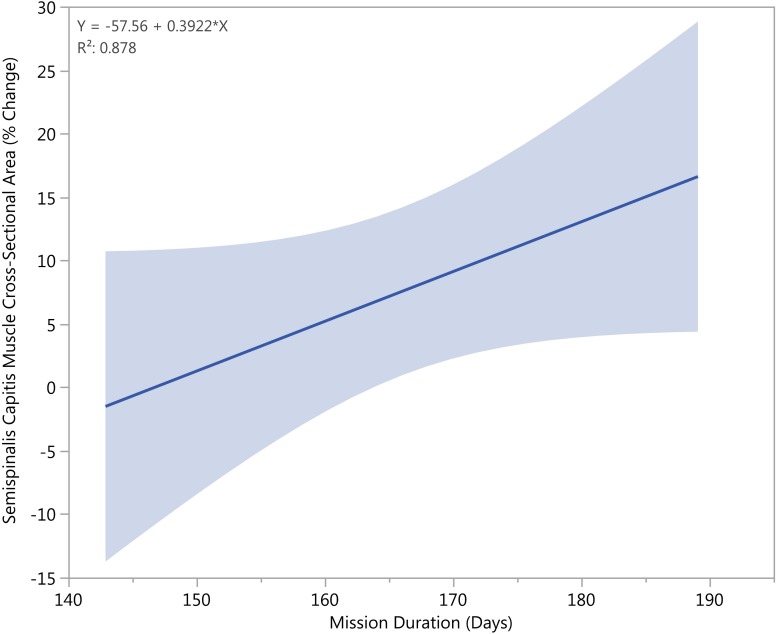
Longer mission durations correlated with increased growth of the semispinalis capitis CSA (*p* < 0.05).

No significant regression equations were found to predict muscle CSA percent change based on the mission duration (*n* = 4, α = 0.05). However, there was a trend toward significance in that crewmembers who spent longer time at the ISS tended to have larger increases in their semispinalis capitis muscle CSA equivalent to roughly a 2% additional increase per every five days of extra time on the ISS (*R*^2^ = 0.88, *p* < 0.1) (Figure 6).

**FIGURE 7:**
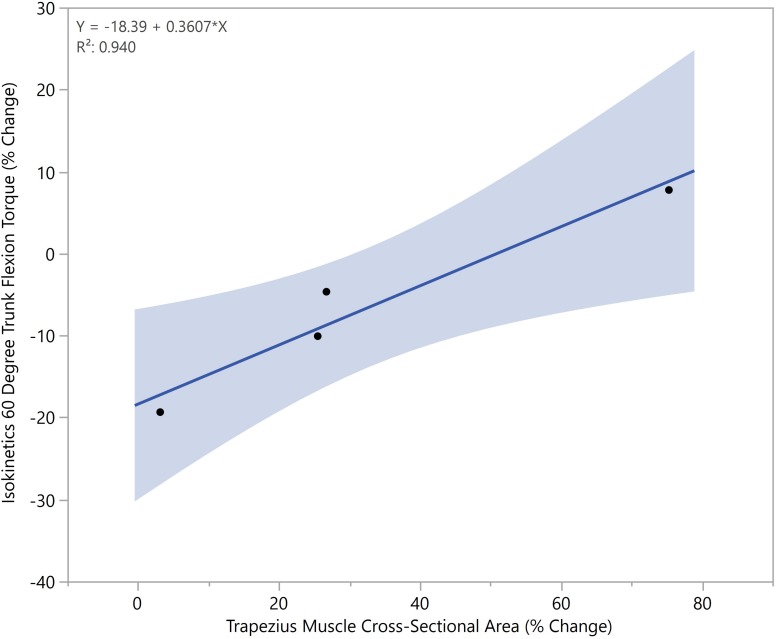
Increased trapezius cross-sectional areas correlated with increased preservation of truncal strength (*p* < 0.05).

No significant regression equation to predict trunk flexion strength based on the changes in muscle CSA was found (*n* = 4, α = 0.05). However, there was a trend toward significance in that crewmembers who had larger increases in their trapezius tended to better preserve their truncal strength compared to their peers. For every 3% increase in trapezius muscle area, a crewmember tended to have an additional 1% preserved truncal strength compared to their peers (*R*^2^ = 0.94, *p* < 0.1) (Figure 7).

There were no other trends seen when comparing muscle changes to mission duration or isokinetic strength changes.

## Discussion

The results of this study support prior studies that found long-duration spaceflight does not lead to atrophy of the neck muscles of astronauts. The study shows the degree to which different neck muscles are affected on current ISS missions.

The novel finding of our study was the increase in the trapezius, semispinalis capitis, rhomboid minor, and sternocleidomastoid CSA. This may be attributed to the constant daily use of these particular neck muscles during spaceflight; however, further studies would be needed to test this hypothesis. It is also possible that the neck muscle hypertrophy occured because the muscles are not as heavily loaded on Earth as lower back and limb musculature, decreasing the potential for atrophy (Belavý et al., 2013). Collectively, these three muscle groups are responsible for the flexion, extension, and torsion movements of the neck that are required for nearly all everyday tasks on the ISS and for maintaining an upright positioning of the head. Furthermore, a lack of gravitational pull could weaken the vestibular nuclei’s sense of balance and require more activity of the neck muscles to move the head in order to take in visual input to compensate. As such, it is reasonable to assume that these muscles are adequately maintained or even strengthened during spaceflight.

These data compare favorably with a volumetric analysis of neck musculature during two months of bed rest with a resistive exercise intervention (Belavý et al., 2013). The semispinalis capitis, longissimus capitis, splenius capitis, levator scapulae, sternocleidomastoid, and the anterior and posterior scalenes all had similar changes or lack thereof. However, the astronaut population had a decrease in posterior scalene size, unlike the bed rest study, although neither study found statistical significance in those results. These comparisons lend credence to the revised hypothesis that lack of spinal loading is driving the enlargement of neck muscles seen in this study since that was the main commonality between the two study setups.

Conditions of decreased muscle strength such as sarcopenia lead to increased mortality and decreased quality of life (Boutin et al., 2015, 2017; Lenchik et al., 2018; Murea et al., 2018). Astronauts are prone to sarcopenia manifesting as increased fatigability and decreased strength of muscles (Thornton and Rummel, 1977; Cruz-Jentoft et al., 2010). Muscle size is just one metric to evaluate muscle strength. Bed rest studies have also shown decreases in type I and type II myofiber CSAs (Bamman et al., 1998). Aging and muscle-wasting diseases have shown a relationship between muscle strength and fat infiltration of muscle (Jones et al., 1983; Liu et al., 1993; Ryan and Nicklas, 1999; Goodpaster et al., 2001; Visser et al., 2002). Investigations into the fat content of neck muscles as well as curvature and intervertebral disc changes may help explain the persistence of herniated nucleus pulposus risk despite preserved muscle size (Johnston et al., 2010; Bailey et al., 2018; Green and Scott, 2018). While MFI was included in this analysis, none of the changes with spaceflight were found to be significant. At this time, the overall results of this study describe the areal changes seen in the neck muscles, but do not explain the persistence of spinal pain and injury in the astronaut population. Further investigation would be necessary to compare these spinal CSA changes to functional changes in the muscles studied.

In-flight countermeasures are thought to aid in the prevention of post-flight injuries (Sayson et al., 2013). However, aRED lacks an exercise protocol that mimics terrestrial cervical spine loading (Sayson et al., 2013), which may explain why spinal pain and herniated discs persist despite the introduction of resistive exercise on ISS missions (Laughlin et al., 2016). NASA’s Astronaut Strength, Conditioning, and Rehabilitation team focuses on the optimization of in-flight exercise regimens as well as post-flight rehabilitation to mitigate the risks of these spinal injuries. While the results of this study demonstrate maintenance of the neck muscles onboard ISS, the risk of spinal injuries persist. The inclusion of in-flight pharmacologic agents could be used to aid in the maintenance of muscle health to offset in-flight musculoskeletal changes and to improve the rates of recovery to baseline values (LeBlanc et al., 2002; Zhang et al., 2011; Smith et al., 2012; Leblanc et al., 2013; Lloyd et al., 2015; MacNabb et al., 2016; Lang et al., 2017; Ramachandran et al., 2018). While many pharmacotherapies such as bisphosphonates, osteoprotegerin, sclerostin antibodies, and parathyroid hormone have been proposed to quell bone loss, a few pharmacotherapies are being studied to prevent muscle atrophy as well (Ramachandran et al., 2018). These include myostatin inhibitors to encourage muscle proliferation and peptide supplements to discourage muscle atrophy (Ramachandran et al., 2018). It is important to continue to quantify the current effects of ISS missions on neck musculature when evaluating the efficacy of in-flight interventions.

## Limitations

Study limitations included a small sample size for both the overall study (*n* = 6), and the sub-study using exercise logs and trunk flexion torque changes (*n* = 4). However, there were only 18 crewmembers on ISS missions during the data collection period. The study was also limited by the preponderance of male ISS crewmembers during the data collection period. Finally, the study was limited by the MRI scan parameters which did not allow for a comprehensive evaluation of MFI. Ideally, studies should include chemical-shift imaging methods such as the Dixon technique to capture water- and fat-only images that can be utilized to more accurately evaluate muscle fat content (Crawford et al., 2016). The available scan data only made it possible to determine atrophy or hypertrophy of a muscle rather than muscle quality or muscle strength. Despite these limitations, statistically significant changes were found in four of the 12 muscles analyzed. Future studies by our research team will address these limitations by actively recruiting a more representative sample and combining both MRI and computed tomography techniques to evaluate both muscle composition and size.

## Conclusion

Using pre- and post-flight MRI scan analysis, we quantified changes in neck musculature in crewmembers of long-duration space missions during the aRED era. None of the muscles showed statistically significant decreases in their CSAs as a result of prolonged microgravity. In addition, post-flight hypertrophy was observed in the semispinalis capitis, sternocleidomastoid, trapezius, and rhomboid minor muscles. Future studies should include assessments of changes in intervertebral disc heights, cervical curvature, and vertebral bone density, and cortical thickness in order to create a more robust picture of how microgravity places astronauts at increased risk for spine injury. Eventually, future studies could allow for in-flight interventions that target musculoskeletal changes and mitigate the risk of injury.

## Data Availability

The datasets for this study will not be made publicly available because MRI scans were obtained from the Life Sciences Data Archive and Lifetime Surveillance of Astronaut Health project.

## Author Contributions

LL, JT, and AW designed the research. KM, KG, JT, JD, and KK performed the data collection, data analysis, and statistical analysis. LL and AW interpreted the data. KM, KG, JT, LL, and AW wrote and revised the manuscript. AW had primary responsibility for final content. All authors listed have made a substantial, direct and intellectual contribution to the work, and approved it for publication.

## Conflict of Interest Statement

The authors declare that the research was conducted in the absence of any commercial or financial relationships that could be construed as a potential conflict of interest.
